# Bis(diiso­butyl­ammonium) tetra­chlorido­bis­[3-(tri­fluorometh­yl)phen­yl]stannate

**DOI:** 10.1107/S2414314623009136

**Published:** 2023-10-24

**Authors:** Xueqing Song, William Li

**Affiliations:** a University of the District of Columbia, Chemistry, 4200 Connecticut Avenue, NW, Washington DC, 20008, USA; Sunway University, Malaysia

**Keywords:** stannate, ammonium cation, crystal structure, hydrogen bonding, salt

## Abstract

The asymmetric unit in the title salt, (C_8_H_20_N)_2_[SnCl_4_(C_7_H_4_Cl_2_F_3_)_2_], features a di-*iso*butyl­ammonium cation in a general position and a diorganotin tetra­chloride dianion, *i.e*. tetra­chlorido­bis­(3-trifuoro­methyl­phen­yl)stannate(IV), located on a centre of inversion; the Sn^IV^ atom is octa­hedrally coordinated. In the crystal, charge-assisted N^+^—H⋯Cl hydrogen bonds along with C—H⋯F contacts occur within supra­molecular layers that inter­digitate along the *a*-axis direction.

## Abstract 2

The title salt comprises di-*iso*butyl­ammonium cations and centrosymmetric tetra­chloro­bis-(3-trifuoro­methyl­phen­yl)stannate(IV) anions, which are connected in the crystal by N—H⋯Cl and C—H⋯F inter­actions.

## Graphical abstract

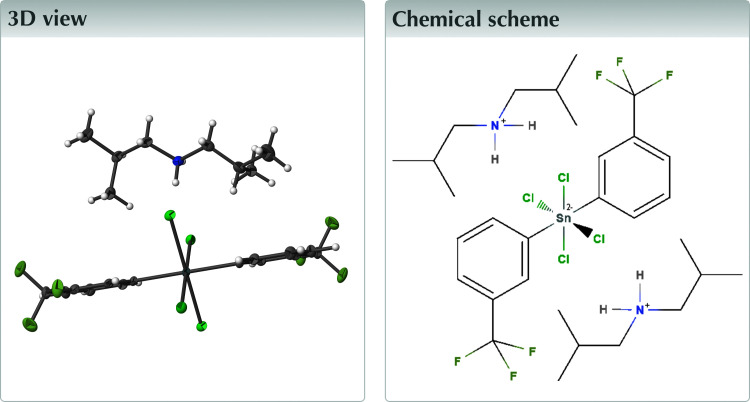

## Structure description

The title salt, bis­(di-*iso*butyl­ammonium) tetra­chlorido­bis­(3-tri­fluoro­methyl­phen­yl)stannate, was obtained as a by-product in a reaction of tris­(3-tri­fluoro­methyl­phen­yl)tin chloride with acetic acid in the presence of di-*iso*butyl­amine. An inter­esting Sn—C cleavage occurred during this reaction.

**Figure 1:**
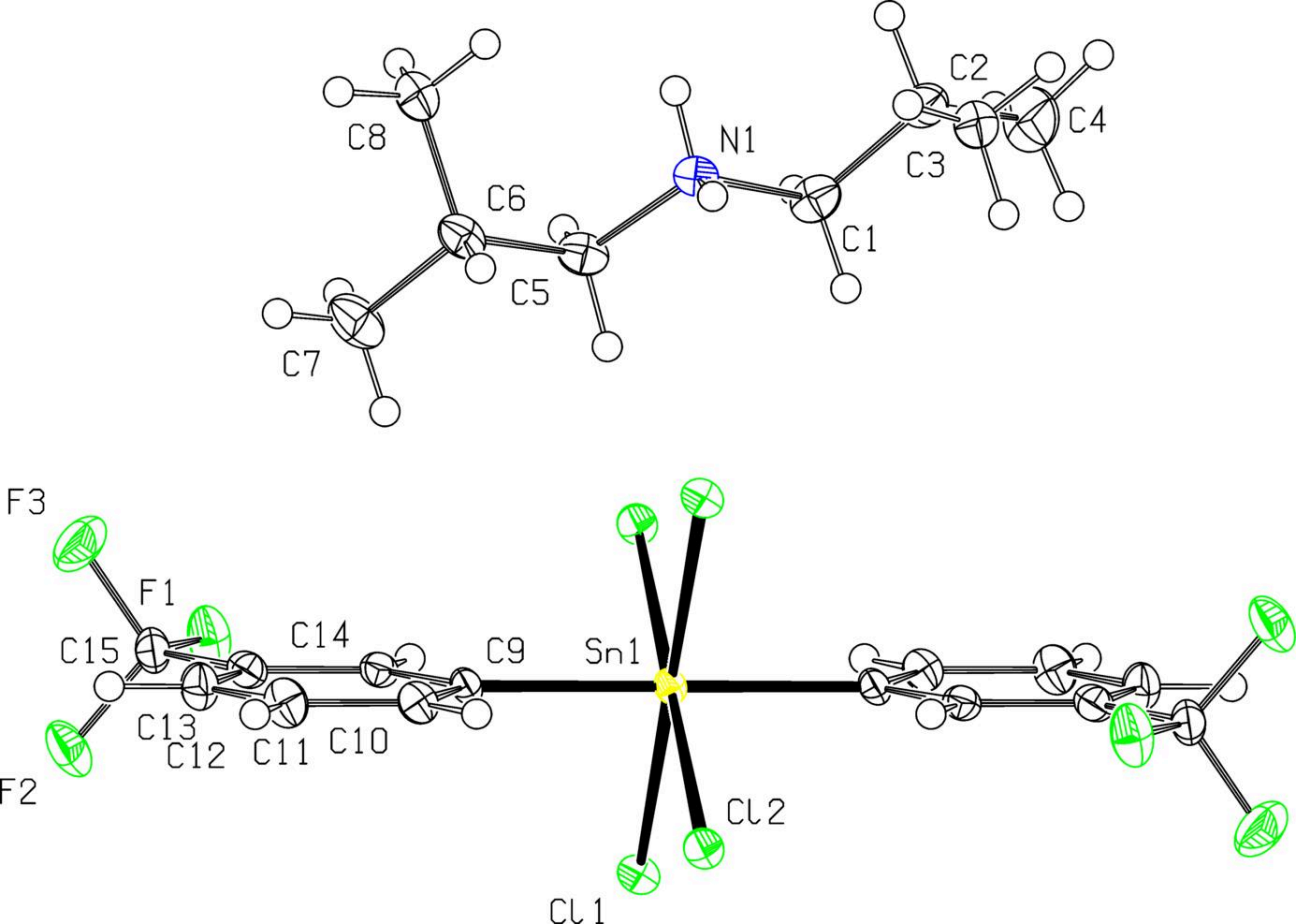
A view of the mol­ecular structures of the di-*iso*butyl­ammonium cation and the tetra­chlorido­bis-(3-trifuoro­methyl­phen­yl)stannate(IV) anion, showing the atom-labelling scheme and displacement ellipsoids at the 50% probability level. The unlabelled atoms for the anion are related by 1 − *x*, 1 − *y*, 1 − *z*.

The crystal comprises di-*iso*butyl­ammonium cations and tetra­chlorido­bis-(3-tri­fluoro­methyl­phen­yl)stannate(IV) anions, with the Sn^IV^ atom of the latter located on a centre of inversion, Fig. 1 ▸. The coordination geometry about the Sn^IV^ atom is based on an octa­hedron, Table 1 ▸. This observation resembles literature precedents, *e.g*. Teoh *et al.* (1992 ▸) and Hazell *et al.* (1998 ▸).

**Figure 2:**
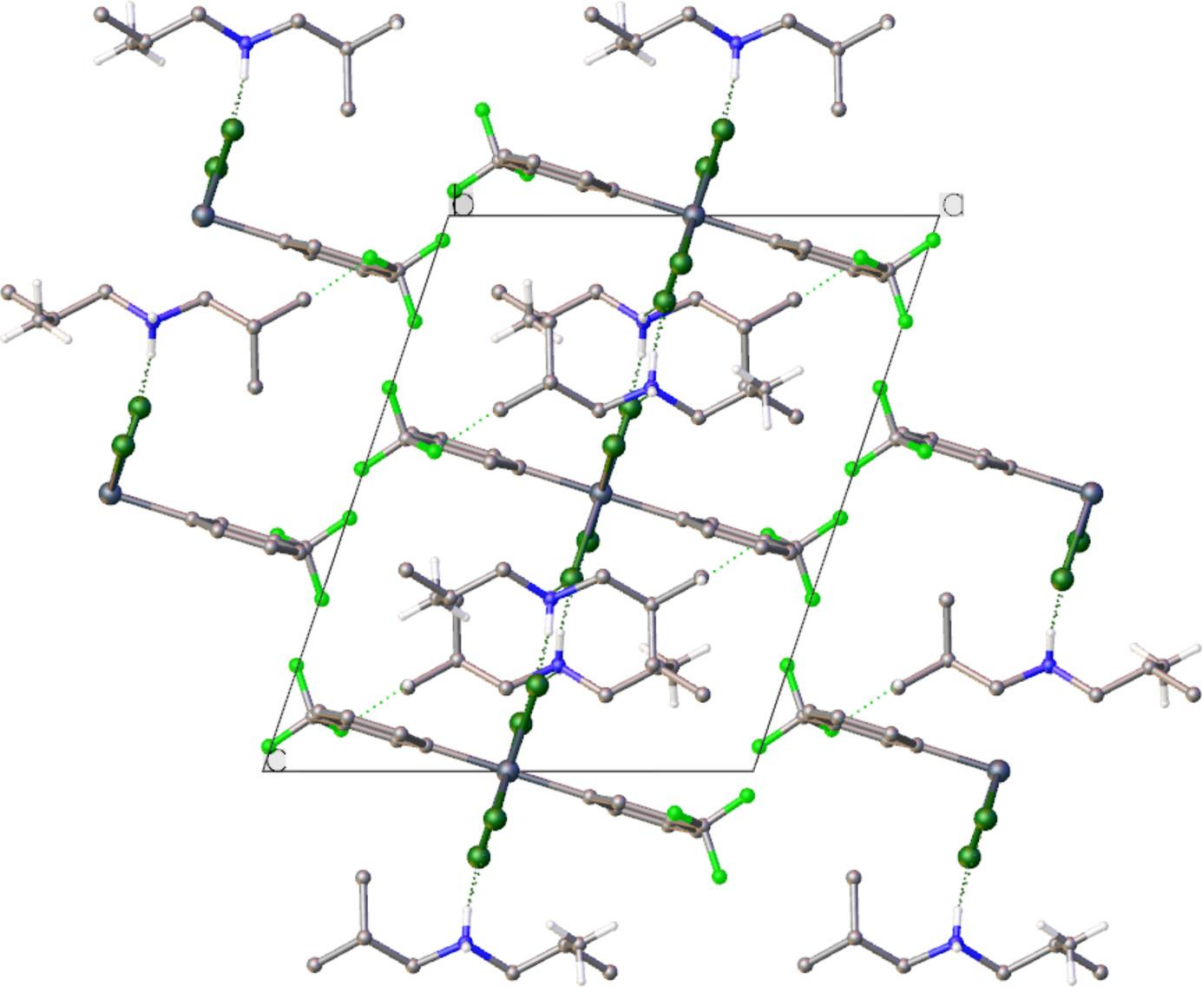
A packing diagram viewed along [010] with inter­molecular hydrogen bonding shown as dashed lines.

In the crystal, charge-assisted N^+^—H⋯Cl hydrogen bonds along with C—H⋯F contacts link mol­ecules into a supra­molecular layer parallel to (011). As noted from Table 2 ▸, the Cll atom accepts two N^+^—H⋯Cl hydrogen bonds, each of which is significantly shorter than the N^+^—H⋯Cl hydrogen bond involving the Cl2 atom. This observation accounts for the disparity in the Sn—Cl bond lengths, Table 1 ▸. The supra­molecular layers interdigitate along [100], Fig. 2 ▸.

## Synthesis and crystallization

The crystal was obtained as a by-product in an attempt to poduce tris­(3-tri­fluoro­methyl­phen­yl)tin acetate in a reaction involving tris­(3-tri­fluoro­methyl­phen­yl)tin chloride and acetic acid in the presence of di-iso­butyl­amine. The anti­cipated tris­(3-tri­fluoro­methyl­phen­yl)tin acetate was isolated as the major product along with a few smaller crystals of the title compound in the mother liquid, comprising a mixture of di­chloro­methane and hexane.

## Refinement

Crystal data, data collection and structure refinement details are summarized in Table 3 ▸.

## Supplementary Material

Crystal structure: contains datablock(s) I. DOI: 10.1107/S2414314623009136/tk4096sup1.cif

Structure factors: contains datablock(s) I. DOI: 10.1107/S2414314623009136/tk4096Isup2.hkl

CCDC reference: 2301782

Additional supporting information:  crystallographic information; 3D view; checkCIF report

## Figures and Tables

**Table 1:** Selected geometric parameters (Å, °)

Sn1—Cl1	2.5845 (4)	Sn1—C9	2.147 (2)
Sn1—Cl2	2.5719 (4)		
			
C9—Sn1—Cl1	89.36 (6)	Cl1—Sn1—Cl2	90.308 (14)
C9—Sn1—Cl2	90.11 (5)		

**Table 2:** Hydrogen-bond geometry (Å, °)

*D*—H⋯*A*	*D*—H	H⋯*A*	*D*⋯*A*	*D*—H⋯*A*
N1—H1*C*⋯Cl1^i^	0.91	2.31	3.1877 (17)	161
N1—H1*D*⋯Cl1	0.91	2.44	3.1771 (17)	138
N1—H1*D*⋯Cl2	0.91	2.75	3.4094 (17)	130
C7—H7*A*⋯F1^ii^	0.98	2.56	3.227 (3)	125

Symmetry codes: (i) 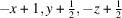 ; (ii) 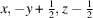 .

**Table 3:** Experimental details

Crystal data	
Chemical formula	(C_8_H_20_N)_2_[SnCl_4_(C_7_H_4_Cl_2_F_3_)_2_]
*M*_r_	811.19
Crystal system, space group	Monoclinic, *P*2_1_/*c*
Temperature (K)	100
*a*, *b*, *c* (Å)	12.2614 (1), 10.8318 (1), 14.6297 (1)
β (°)	108.523 (1)
*V* (Å^3^)	1842.36 (3)
*Z*	2
Radiation type	Cu *K*α
μ (mm^−1^)	8.64
Crystal size (mm)	0.1 × 0.07 × 0.03

Data collection	
Diffractometer	XtaLAB Synergy, Single source at home/near, HyPix
Absorption correction	Multi-scan (*CrysAlis PRO*; Rigaku OD, 2023 ▸)
*T*_min_, *T*_max_	0.293, 1.000
No. of measured, independent and observed [*I* > 2σ(*I*)] reflections	18706, 3822, 3652
*R*_int_	0.057
(sin θ/λ)_max_ (Å^−1^)	0.634

Refinement	
*R*[*F* ^2^ > 2σ(*F* ^2^)], *wR*(*F* ^2^), *S*	0.027, 0.075, 1.08
No. of reflections	3822
No. of parameters	200
H-atom treatment	H-atom parameters constrained
Δρ_max_, Δρ_min_ (e Å^−3^)	0.66, −0.90

Computer programs: *CrysAlis PRO* (Rigaku OD, 2023 ▸), *SHELXT2018/2* (Sheldrick, 2015*a* ▸), *SHELXL2018/3* (Sheldrick, 2015*b* ▸) and *OLEX2* (Dolomanov *et al.*, 2009 ▸).

## full crystallographic data

### Crystal data

**Table:**

(C_8_H_20_N)_2_[SnCl_4_(C_7_H_4_Cl_2_F_3_)_2_]	*F*(000) = 828
*M**_r_* = 811.19	*D*_x_ = 1.462 Mg m^−^^3^
Monoclinic, *P*2_1_/*c*	Cu *K*α radiation, λ = 1.54184 Å
*a* = 12.2614 (1) Å	Cell parameters from 14287 reflections
*b* = 10.8318 (1) Å	θ = 3.8–77.8°
*c* = 14.6297 (1) Å	µ = 8.64 mm^−^^1^
β = 108.523 (1)°	*T* = 100 K
*V* = 1842.36 (3) Å^3^	Block, clear brown-orange
*Z* = 2	0.1 × 0.07 × 0.03 mm

### Data collection

**Table:**

XtaLAB Synergy, Single source at home/near, HyPix diffractometer	3652 reflections with *I* > 2σ(*I*)
Detector resolution: 10.0000 pixels mm^-1^	*R*_int_ = 0.057
ω scans	θ_max_ = 78.0°, θ_min_ = 3.8°
Absorption correction: multi-scan (CrysAlisPro; Rigaku OD, 2023)	*h* = −15→15
*T*_min_ = 0.293, *T*_max_ = 1.000	*k* = −13→11
18706 measured reflections	*l* = −18→17
3822 independent reflections	

### Refinement

**Table:**

Refinement on *F*^2^	0 restraints
Least-squares matrix: full	Hydrogen site location: inferred from neighbouring sites
*R*[*F*^2^ > 2σ(*F*^2^)] = 0.027	H-atom parameters constrained
*wR*(*F*^2^) = 0.075	*w* = 1/[σ^2^(*F*_o_^2^) + (0.041*P*)^2^ + 0.7503*P*] where *P* = (*F*_o_^2^ + 2*F*_c_^2^)/3
*S* = 1.08	(Δ/σ)_max_ < 0.001
3822 reflections	Δρ_max_ = 0.66 e Å^−^^3^
200 parameters	Δρ_min_ = −0.90 e Å^−^^3^

### Special details

**Table:**

Geometry. All esds (except the esd in the dihedral angle between two l.s. planes) are estimated using the full covariance matrix. The cell esds are taken into account individually in the estimation of esds in distances, angles and torsion angles; correlations between esds in cell parameters are only used when they are defined by crystal symmetry. An approximate (isotropic) treatment of cell esds is used for estimating esds involving l.s. planes.

### Fractional atomic coordinates and isotropic or equivalent isotropic displacement parameters (Å^2^)

**Table:**

	*x*	*y*	*z*	*U*_iso_*/*U*_eq_	
C1	0.35270 (19)	0.5478 (2)	0.13703 (15)	0.0239 (4)	
H1A	0.358554	0.457309	0.130324	0.029*	
H1B	0.326308	0.583776	0.071441	0.029*	
C2	0.26367 (19)	0.5751 (2)	0.18694 (15)	0.0260 (5)	
H2	0.295430	0.546221	0.255225	0.031*	
C3	0.23739 (19)	0.7127 (2)	0.18845 (16)	0.0275 (5)	
H3A	0.306651	0.756553	0.226965	0.041*	
H3B	0.175849	0.724982	0.216927	0.041*	
H3C	0.212799	0.744875	0.122425	0.041*	
C4	0.1551 (3)	0.5004 (2)	0.1380 (3)	0.0411 (8)	
H4A	0.121094	0.528525	0.071310	0.062*	
H4B	0.099624	0.511991	0.172948	0.062*	
H4C	0.174867	0.412669	0.138369	0.062*	
C5	0.56054 (19)	0.55525 (19)	0.14964 (15)	0.0222 (4)	
H5A	0.537390	0.577720	0.080518	0.027*	
H5B	0.565458	0.464099	0.154047	0.027*	
C6	0.67905 (19)	0.60917 (19)	0.20024 (15)	0.0218 (4)	
H6	0.674857	0.700698	0.190829	0.026*	
C7	0.7618 (2)	0.5571 (2)	0.15105 (19)	0.0328 (5)	
H7A	0.765195	0.467094	0.157844	0.049*	
H7B	0.838600	0.591970	0.181228	0.049*	
H7C	0.734647	0.578953	0.082497	0.049*	
C8	0.72259 (19)	0.5828 (2)	0.30827 (16)	0.0274 (5)	
H8A	0.669735	0.619158	0.338881	0.041*	
H8B	0.799278	0.618867	0.336388	0.041*	
H8C	0.726737	0.493329	0.318920	0.041*	
N1	0.46944 (15)	0.59824 (15)	0.19037 (12)	0.0177 (3)	
H1C	0.466464	0.682178	0.188412	0.021*	
H1D	0.488962	0.574815	0.253256	0.021*	
C9	0.68457 (19)	0.50380 (16)	0.54647 (16)	0.0146 (4)	
C10	0.74444 (18)	0.6150 (2)	0.56702 (15)	0.0178 (4)	
H10	0.702813	0.690201	0.560520	0.021*	
C11	0.86397 (19)	0.6180 (2)	0.59687 (16)	0.0220 (4)	
H11	0.903295	0.694773	0.610510	0.026*	
C12	0.9258 (2)	0.50866 (18)	0.60672 (18)	0.0215 (5)	
H12	1.007476	0.509922	0.627119	0.026*	
C13	0.86647 (18)	0.3971 (2)	0.58629 (14)	0.0180 (4)	
C14	0.74712 (18)	0.39424 (19)	0.55628 (14)	0.0157 (4)	
H14	0.707913	0.317433	0.542374	0.019*	
C15	0.93450 (17)	0.2801 (2)	0.59848 (15)	0.0205 (4)	
Cl1	0.50101 (4)	0.38477 (4)	0.34558 (3)	0.01643 (11)	
Cl2	0.49391 (4)	0.70843 (4)	0.41432 (3)	0.01725 (11)	
F1	0.86867 (11)	0.17902 (12)	0.57535 (11)	0.0291 (3)	
F2	1.00453 (12)	0.27762 (13)	0.54416 (10)	0.0306 (3)	
F3	1.00321 (13)	0.26451 (14)	0.69011 (10)	0.0364 (3)	
Sn1	0.500000	0.500000	0.500000	0.01258 (8)	

### Atomic displacement parameters (Å^2^)

**Table:**

	*U*^11^	*U*^22^	*U*^33^	*U*^12^	*U*^13^	*U*^23^
C1	0.0260 (11)	0.0198 (11)	0.0220 (9)	−0.0067 (9)	0.0021 (8)	−0.0019 (8)
C2	0.0229 (10)	0.0294 (11)	0.0233 (10)	−0.0069 (9)	0.0038 (8)	0.0065 (9)
C3	0.0221 (11)	0.0338 (13)	0.0268 (10)	0.0007 (9)	0.0080 (8)	0.0042 (9)
C4	0.0291 (15)	0.0419 (19)	0.0480 (18)	−0.0161 (10)	0.0061 (13)	0.0051 (10)
C5	0.0301 (11)	0.0173 (10)	0.0218 (9)	0.0001 (8)	0.0118 (8)	−0.0026 (8)
C6	0.0270 (10)	0.0160 (9)	0.0269 (10)	0.0026 (8)	0.0151 (8)	0.0001 (8)
C7	0.0383 (13)	0.0285 (13)	0.0404 (13)	0.0049 (10)	0.0249 (11)	−0.0024 (10)
C8	0.0212 (10)	0.0331 (12)	0.0300 (11)	−0.0014 (9)	0.0112 (8)	0.0022 (10)
N1	0.0220 (8)	0.0135 (8)	0.0181 (7)	−0.0022 (6)	0.0071 (6)	−0.0008 (6)
C9	0.0117 (10)	0.0175 (11)	0.0161 (10)	−0.0006 (6)	0.0063 (8)	−0.0003 (6)
C10	0.0190 (10)	0.0152 (10)	0.0204 (9)	0.0002 (8)	0.0081 (7)	−0.0026 (8)
C11	0.0199 (10)	0.0188 (11)	0.0275 (10)	−0.0063 (8)	0.0077 (8)	−0.0041 (8)
C12	0.0155 (11)	0.0232 (12)	0.0254 (11)	−0.0015 (7)	0.0057 (9)	0.0001 (7)
C13	0.0189 (9)	0.0194 (10)	0.0176 (9)	0.0016 (8)	0.0086 (7)	0.0013 (8)
C14	0.0170 (9)	0.0158 (10)	0.0155 (8)	0.0007 (7)	0.0070 (7)	0.0017 (7)
C15	0.0136 (9)	0.0234 (10)	0.0250 (9)	0.0015 (8)	0.0068 (7)	0.0033 (8)
Cl1	0.0205 (2)	0.0118 (2)	0.0188 (2)	−0.00014 (15)	0.00893 (16)	−0.00113 (15)
Cl2	0.0188 (2)	0.0129 (2)	0.0212 (2)	0.00085 (15)	0.00785 (16)	0.00294 (16)
F1	0.0222 (6)	0.0172 (6)	0.0500 (8)	0.0021 (5)	0.0143 (6)	0.0042 (6)
F2	0.0271 (7)	0.0289 (7)	0.0442 (8)	0.0067 (5)	0.0235 (6)	0.0043 (6)
F3	0.0351 (8)	0.0389 (8)	0.0284 (6)	0.0168 (6)	0.0005 (6)	0.0061 (6)
Sn1	0.01231 (11)	0.01048 (12)	0.01599 (11)	0.00097 (5)	0.00599 (8)	0.00084 (5)

### Geometric parameters (Å, º)

**Table:**

C1—C2	1.523 (3)	C11—C12	1.389 (3)
C1—N1	1.499 (3)	C12—C13	1.393 (3)
C2—C3	1.527 (3)	C13—C14	1.388 (3)
C2—C4	1.528 (3)	C13—C15	1.497 (3)
C5—C6	1.522 (3)	C15—F1	1.338 (3)
C5—N1	1.497 (3)	C15—F2	1.343 (2)
C6—C7	1.526 (3)	C15—F3	1.348 (2)
C6—C8	1.526 (3)	Sn1—Cl1	2.5845 (4)
C9—C10	1.393 (3)	Sn1—Cl2	2.5719 (4)
C9—C14	1.396 (3)	Sn1—C9	2.147 (2)
C10—C11	1.390 (3)		
			
N1—C1—C2	113.03 (17)	F1—C15—F2	106.35 (17)
C1—C2—C3	112.52 (18)	F1—C15—F3	106.55 (17)
C1—C2—C4	108.9 (2)	F2—C15—C13	112.55 (17)
C3—C2—C4	111.5 (2)	F2—C15—F3	105.72 (16)
N1—C5—C6	113.87 (16)	F3—C15—C13	111.92 (17)
C5—C6—C7	107.69 (18)	C9—Sn1—C9^i^	180.0
C5—C6—C8	113.43 (18)	C9^i^—Sn1—Cl1^i^	89.36 (6)
C7—C6—C8	110.67 (19)	C9—Sn1—Cl1	89.36 (6)
C5—N1—C1	112.90 (16)	C9—Sn1—Cl1^i^	90.64 (6)
C10—C9—C14	118.6 (2)	C9^i^—Sn1—Cl1	90.64 (6)
C10—C9—Sn1	121.02 (14)	C9—Sn1—Cl2^i^	89.89 (5)
C14—C9—Sn1	120.40 (14)	C9^i^—Sn1—Cl2^i^	90.11 (5)
C11—C10—C9	121.3 (2)	C9^i^—Sn1—Cl2	89.89 (5)
C12—C11—C10	119.9 (2)	C9—Sn1—Cl2	90.11 (5)
C11—C12—C13	119.1 (2)	Cl1^i^—Sn1—Cl1	180.0
C12—C13—C15	118.4 (2)	Cl1—Sn1—Cl2	90.308 (14)
C14—C13—C12	120.9 (2)	Cl2^i^—Sn1—Cl1^i^	90.308 (14)
C14—C13—C15	120.68 (19)	Cl2^i^—Sn1—Cl1	89.692 (14)
C13—C14—C9	120.20 (19)	Cl2—Sn1—Cl1^i^	89.693 (14)
F1—C15—C13	113.22 (17)	Cl2^i^—Sn1—Cl2	180.00 (3)
			
C2—C1—N1—C5	171.26 (17)	C12—C13—C14—C9	−0.3 (3)
C6—C5—N1—C1	177.79 (17)	C12—C13—C15—F1	−178.98 (19)
N1—C1—C2—C3	66.3 (2)	C12—C13—C15—F2	−58.3 (3)
N1—C1—C2—C4	−169.55 (19)	C12—C13—C15—F3	60.6 (3)
N1—C5—C6—C7	179.98 (17)	C14—C9—C10—C11	−0.1 (3)
N1—C5—C6—C8	57.2 (2)	C14—C13—C15—F1	1.7 (3)
C9—C10—C11—C12	−0.1 (3)	C14—C13—C15—F2	122.3 (2)
C10—C9—C14—C13	0.2 (3)	C14—C13—C15—F3	−118.8 (2)
C10—C11—C12—C13	0.0 (4)	C15—C13—C14—C9	179.09 (19)
C11—C12—C13—C14	0.1 (4)	Sn1—C9—C10—C11	−179.81 (16)
C11—C12—C13—C15	−179.2 (2)	Sn1—C9—C14—C13	179.96 (15)

Symmetry code: (i) −*x*+1, −*y*+1, −*z*+1.

### Hydrogen-bond geometry (Å, º)

**Table:**

*D*—H···*A*	*D*—H	H···*A*	*D*···*A*	*D*—H···*A*
N1—H1*C*···Cl1^ii^	0.91	2.31	3.1877 (17)	161
N1—H1*D*···Cl1	0.91	2.44	3.1771 (17)	138
N1—H1*D*···Cl2	0.91	2.75	3.4094 (17)	130
C7—H7*A*···F1^iii^	0.98	2.56	3.227 (3)	125

Symmetry codes: (ii) −*x*+1, *y*+1/2, −*z*+1/2; (iii) *x*, −*y*+1/2, *z*−1/2.
